# “Late-stage” deforestation enhances storm trends in coastal West Africa

**DOI:** 10.1073/pnas.2109285119

**Published:** 2022-01-04

**Authors:** Christopher M. Taylor, Cornelia Klein, Douglas J. Parker, France Gerard, Valiyaveetil Shamsudheen Semeena, Emma J. Barton, Bethan L. Harris

**Affiliations:** ^a^UK Centre for Ecology and Hydrology, Wallingford OX10 8BB, United Kingdom;; ^b^National Centre for Earth Observation, Wallingford OX10 8BB, United Kingdom;; ^c^Department of Atmospheric and Cryospheric Sciences, University of Innsbruck, 6020 Innsbruck, Austria;; ^d^Institute for Climate and Atmospheric Science, University of Leeds, Leeds LS2 9JT, United Kingdom

**Keywords:** deforestation, West Africa, convective storms, rainfall, sea breeze

## Abstract

Deforestation affects local and regional hydroclimate through changes in heating and moistening of the atmosphere. In the tropics, deforestation leads to warming, but its impact on rainfall is more complex, as it depends on spatial scale and synoptic forcing. Most studies have focused on Amazonia, highlighting that forest edges locally enhance convective rainfall, whereas rainfall decreases over drier, more extensive, deforested regions. Here, we examine Southern West Africa (SWA), an example of “late-stage” deforestation, ongoing since 1900 within a 300-km coastal belt. From three decades of satellite data, we demonstrate that the upward trend in convective activity is strongly modulated by deforestation patterns. The frequency of afternoon storms is enhanced over and downstream of deforested patches on length scales from 16 to 196 km, with greater increases for larger patches. The results are consistent with the triggering of storms by mesoscale circulations due to landscape heterogeneity. Near the coast, where sea breeze convection dominates the diurnal cycle, storm frequency has doubled in deforested areas, attributable to enhanced land–sea thermal contrast. These areas include fast-growing cities such as Freetown and Monrovia, where enhanced storm frequency coincides with high vulnerability to flash flooding. The proximity of the ocean likely explains why ongoing deforestation across SWA continues to increase storminess, as it favors the impact of mesoscale dynamics over moisture availability. The coastal location of deforestation in SWA is typical of many tropical deforestation hotspots, and the processes highlighted here are likely to be of wider global relevance.

## Significance

Tropical deforestation tends to increase regional air temperatures, but its impacts on rainfall are more complex. The conventional picture, based largely on studies over Amazonia, is that storm frequency can increase over small, deforested areas but is reduced when the landscape is predominantly deforested. This study examines Southern West Africa, a coastal region that has little remaining intact forest. Here, the ongoing patchy deforestation increases the frequency of afternoon storms locally. Deforestation appears especially effective at triggering storms near the coast, where temperature-sensitive sea breezes dominate, and rapidly urbanizing populations are vulnerable to flood risk. Our results emphasize dynamical processes over moisture limitation and are highly relevant for many tropical deforestation hotspots, which, unlike Amazonia, are located near the coastline.

Deforestation is dramatically changing the tropical landscape in hotspots within Amazonia (1) and across Southeast Asia and Africa (2). Deforestation can strongly impact regional climate via changes in land–atmosphere fluxes of heat, moisture, and momentum (3, 4, 5). Forests typically have higher leaf area index, deeper roots, lower albedo, and are aerodynamically rougher than their surroundings. Relative to deforested land, tropical forests exhibit increased evaporation and reduced sensible heat, particularly during the dry season (6). In response to these surface flux perturbations, pan-tropical analysis has shown that deforestation tends to increase daytime air temperatures across all seasons (7).

How deforestation affects rainfall is a more complex question, with the spatial extent of change being a key control (8). Where there is forest loss over hundreds of kilometers, reduced atmospheric moisture from transpiration suppresses rainfall downstream (9). Deforestation on these scales can also affect large-scale circulations and, hence, moisture advection into a region (10). Where mesoscale deforestation locally enhances sensible heat flux, daytime circulations develop, favoring convective initiation (11–13), as observed in Amazonia during the dry season (14–17) and in West African shrubland (18). The expansion of deforested patches to ∼100 km over recent decades in Amazonia has affected the relationship between rainfall changes and the underlying land cover. While the edges of these large patches continue to trigger convection, the interior of the deforested zone experiences reduced rainfall in response to less moisture (14, 19), an effect expected to dominate as deforestation expands further (3). Contrasts in surface roughness may also impact storms there through providing favored convergence zones at the downwind side of larger deforested patches (19).

**Fig. 1.**
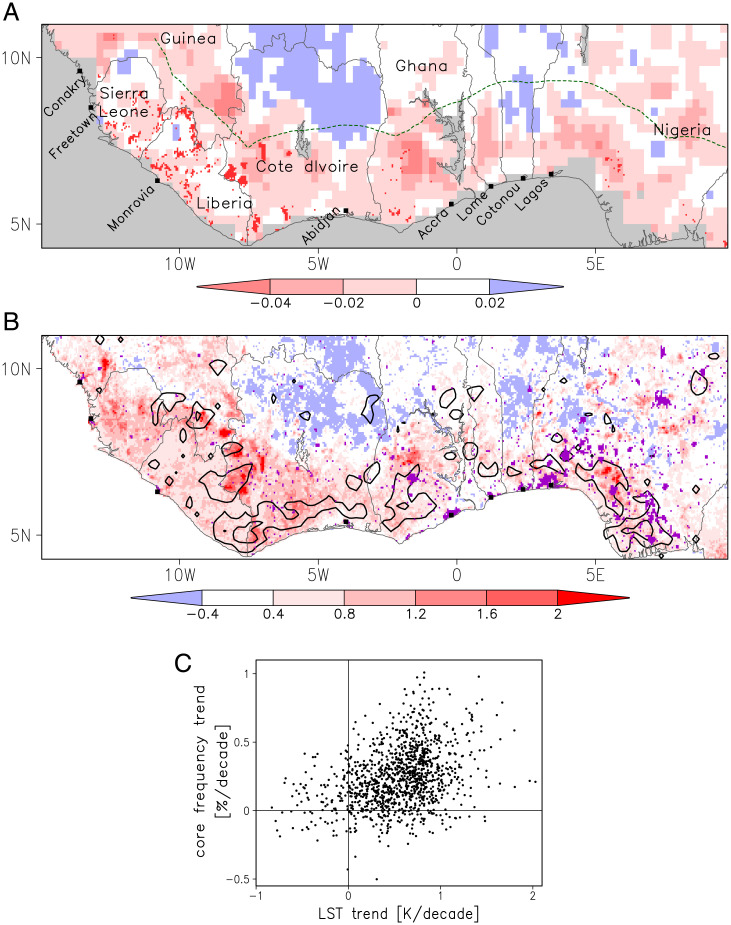
Regional deforestation and trends in convective cores. (*A*) Trends in VOD for 1991 through 2015 (per decade; shading) and forest cover loss exceeding 25% for 2000 to 2019 (dark red pixels). (*B*) LST trend (Kelvin per decade) based on January to February data (1991 to 2021) averaged across Meteosat, Terra, and Aqua (shading). Black contours indicate the 1991 to 2020 trend in core frequency (March to November) sampled at the local diurnal peak. Contour values 0.4 and 0.8% decade^−1^ only plotted where *P* < 0.05. In *A*, the dashed green line denotes 300 km from the coast, and in *B*, purple pixels signify a 10% expansion of settlements. (*C*) Trend in core frequency versus trend in LST (as in *B*) for pixels within 300 km of the coast.

Knowledge of tropical deforestation impacts on rainfall is heavily skewed toward Amazonia, exploiting long-term observational datasets (1). Rainfall responses in other less well-observed deforestation hotspots may vary because of differences in the nature and seasonality of rain-bearing storms, the extent of remaining forest, and the flux characteristics of the deforested landscape. The majority of 21st century tropical deforestation has occurred less than 400 km from the ocean (*SI Appendix*, Fig. 1), with ∼15% within 50 km of the coast. In such locations, the daily advection of humid oceanic air may reduce the influence of evapotranspiration on rainfall, while deforestation could also influence sea breezes through warming of the land.

**Fig. 2.**
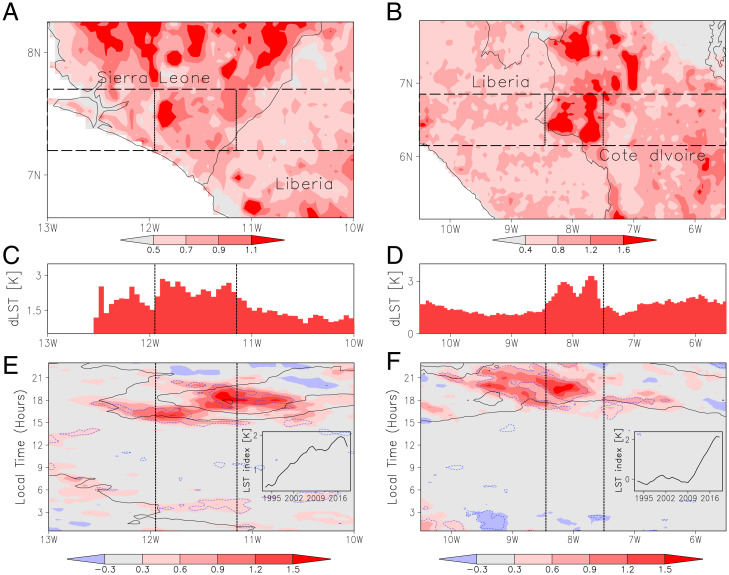
Examples of localized changes in convection with deforestation. LST trends (1991 to 2020; Kelvin per decade) in Sierra Leone (*A*) and Côte d’Ivoire (*B*). Gradients resulting from linear regression of each hotspot’s normalized index with LST (*C* and *D*; Kelvin) and convective core frequency (*E* and *F*; percent) are presented as a function of longitude. The data were averaged between the horizontal lines in *A* and *B* before regressing. In *E* and *F*, data are shown as a function of local time, solid contours depict mean core frequency (percent) for the baseline period 1982 to 1990, and dashed blue lines indicate where the gradients are significantly different from 0 at the 95% level. (*E* and *F*, *Inset*s) An annual index (Kelvin) is computed from within-region LST contrasts to indicate the evolution of deforestation within the central rectangles in *A* and *B*.

This study examines the impact of deforestation on convective storms in Southern West Africa (SWA). Tropical forests in SWA are restricted to zones of high annual rainfall (∼1,000 to 2,500 mm) that extend from the south coast to several hundred kilometers inland. In contrast to Amazonia, significant deforestation has been ongoing since the turn of the 20th century (20), with the majority of the region now occupied by degraded forest, savanna, and agriculture [ref. 21; *SI Appendix*, Fig. 2]. We term this “late-stage deforestation,” a scenario which the majority of Amazonia is not expected to reach for some time. Recent rapid losses of tree cover (Fig. 1*A*) have been linked to conflict (22), cocoa production (23), and rising population (24), the latter concentrated in coastal cities. Thus, densely populated areas are often located close to deforestation hotspots. Any deforestation impacts on convective storms will therefore alter the exposure of urban populations and infrastructure to increased runoff generation and flash floods.

There are two rainy seasons along the south coast of SWA (March to June and September to November), with a “little dry season” in July and August. The little dry season coincides with the peak of the West African monsoon, which brings heavy rain further north in West Africa. Interannual variability in rainfall is strongly influenced by sea surface temperatures (SSTs) in the Tropical Atlantic (25). In contrast to Amazonia, most of the rain comes from mesoscale convective systems (MCSs; ref. 26), which are typically triggered in the afternoon and propagate westwards. There are important, large-scale, positive trends in intense MCSs in SWA (27). More intense African MCSs have been linked to increasing meridional temperature gradients (27–29), while recent decades have seen more frequent MCSs across SWA in the second rainy season (27). Positive trends are also evident for extreme, daily rainfall totals in gauge-based, regional analyses over SWA (30, 31). At the same time, there is a negative trend in total SWA rainfall in the little dry and second rainy seasons, which may be linked to rainfall suppression by anthropogenic aerosols (25). Here, however, we focus on spatial variations in the trends of convective systems on subregional scales and their links to local deforestation.

We use observations of deep convective storms based on half-hourly, cloud-top temperature images, taken from the Meteosat satellite series (available from 1982 onwards). These provide a stable, long-term dataset (29) more suitable for our trend analysis than available precipitation estimates (see *Methods*). We use cloud-top temperature data from March to November (covering the rainy months across the entire region) and apply a wavelet-based technique (32, 33) to identify locally cold cloud features (25 to 50 km), hereafter termed convective cores. This method often filters out extensive, cold cloud areas, which are not associated with convective rain, while retaining the coldest features, which produce the most intense rainfall. We find that 64% of all intense rainfall rates (>20 mm ⋅ h^−1^) occur within the cores (*SI Appendix*, Fig. 5). Moreover, there is a robust correlation (*P* < 0.001) between convective core and precipitation trends across the region when considering a time of day with sufficient, high-quality (HQ) precipitation estimates (*SI Appendix*, Fig. 7).

To map evolving land-use change across the region, we exploit the strong land surface temperature (LST) response to land cover (7). Postdeforestation land has reduced roughness and, particularly during the dry season, is strongly soil moisture limited. Both of these effects increase LST. We use LST data from the dry season months of January and February, coinciding with maximum soil water stress and minimum cloud cover. Climate records of LST date back to 1991 over Africa (34), so we consider trends in LST and convective cores from 1991 to 2020. We preferred this approach to using land-cover classifications, which either did not extend back beyond 2000 (2) or did not provide a consistent annual time series over the Meteosat era.

## Results

**Fig. 3.**
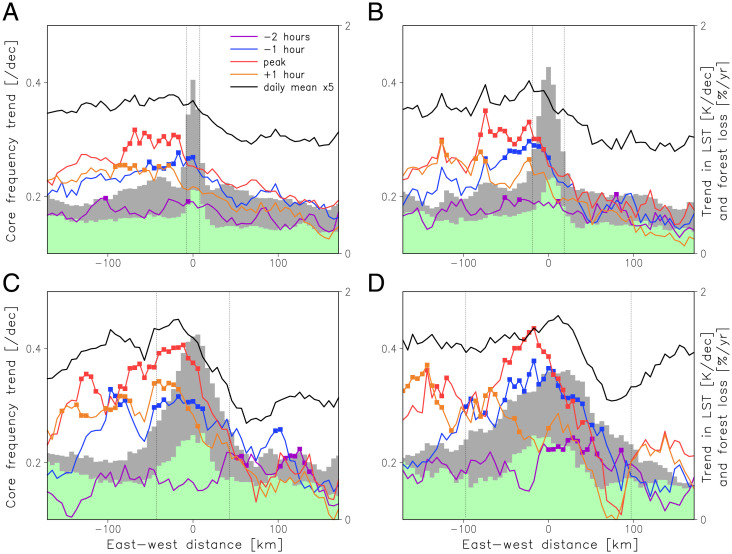
Convection trends composited on deforestation features of different length scales. Composite mean linear trends (1991 to 2020) in convective core frequency (per decade) sampled at different hours relative to the local diurnal peak (colored lines). Squares denote where these trends differ significantly at the 90% level from the zonal mean trend. The 24-h mean trend in core frequency is shown in black and has been multiplied by 5 for clarity. Gray shading denotes the LST trend (1991 to 2021; Kelvin per decade) on which the compositing is based for length scales (indicated by vertical dashed lines) of 16 (*A*), 37 (*B*), 84 (*C*), and 194 km (*D*). Green shading depicts the composite mean forest loss trend (2000 to 2019).

Much of SWA has experienced reductions in woody cover since 1991, as inferred from vegetation optical depth (VOD) trends [Fig. 1*A*; (24)]. In parts of the interior, there are also weak, positive VOD trends, thought to be a response of the ecosystem to increasing rainfall (24). High-resolution tree cover data, available since 2000, show discrete mesoscale pockets of strong deforestation in formerly protected areas (2). The deforestation patterns in both datasets are well captured by LST trends (Fig. 1*B*). Contrasting LST tendencies can be seen either side of national borders, most notably across the frontier zones of still largely forested Liberia and neighbors Côte d’Ivoire and Sierra Leone. However, deforestation is not the only driver of LST trends. At small scales, urbanization strongly increases LST (*SI Appendix*, Fig. 3), affecting parts of Southern Nigeria in particular. At the largest scales, global warming is slowly raising LST. For our LST-based study of deforestation, we exclude pixels containing expanding urban areas (*Methods*) and filter out the global warming signal by only considering pixels with locally strong LST trends. Additional comparisons with VOD, tree cover loss and land use/land cover (LULC) (see *Methods* and *SI Appendix*, Fig. 3) provide a solid justification for our use of LST trends as a proxy for deforestation.

**Fig. 4.**
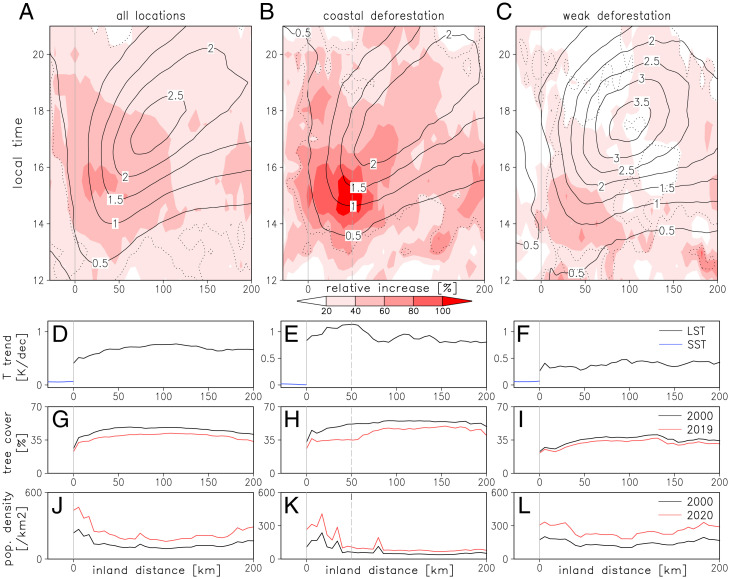
Deforestation influences on sea breeze convection. (*A*–*C*) Composite core frequencies associated with the sea breeze for all coastal locations (*Left*), pixels with strong coastal deforestation (*Middle*), and weak deforestation (*Right*). Shading depicts the relative increase in core frequency (trend/mean × 100%) as a function of distance from coast (x) and local time (y), solid contours indicate the mean core frequency (percent) for the baseline period 1982 to 1990, and dotted contours highlight where the trends are significant (*P* < 0.05). (*D*–*F*) Composite mean LST and SST trends (Kelvin per decade). (*G*–*I*) Composite mean tree cover (percent) in 2000 (black) and 2019 (red). (*J*–*L*) Composite mean population densities (per square kilometer) in 2000 (black) and 2020 (red). In the *Middle*, the coastal strip used to identify locations with strong deforestation lies between the two vertical lines.

To focus on deforestation effects, we examine trends in convection sampled at the local afternoon core frequency maximum (*SI Appendix*, Fig. 4). These trends are largely positive across SWA (Fig. 1 *B* and *C*), consistent with earlier large-scale studies (27, 29). There are, however, strong within-region variations, with a tendency for areas with weak LST trends to have weaker trends in convective activity (Fig. 1*C*; *P* < 0.001). This suggests that deforestation may increase local convective activity during the afternoon and evening period, as observed in Amazonia (14, 16, 19).

Fig. 2 illustrates the evolution of convection in the vicinity of two deforestation hotspots, chosen for both their well-defined spatial pattern and weak orographic forcing. A hotspot LST index has been defined for each region, based on the differential warming of deforested and forested pixels (see *Methods*), which provides an annual proxy time series for deforested area. The indices (Fig. 2 *E* and *F*, *Insets*) illustrate ongoing deforestation in Sierra Leone (compared to Liberia) from the 1990s onwards and the rapid removal of trees starting around 2010 in southwestern Côte d’Ivoire. To assess how the deforestation influences the diurnal evolution of convection at the mesoscale, we regressed the annual time series of meridional mean core frequency for each 30-min period in the day against the hotspot LST index (Fig. 2 *E* and *F*). In each case, we see a strong increase in core frequency develop during late afternoon in the vicinity of maximum deforestation. In the 3 h prior to the diurnal convective maximum, the increase in core frequency can exceed 100% of values observed in the baseline period. This implies that there are more afternoon storm initiations over deforested areas than over neighboring regions. In Côte d’Ivoire, the enhanced convection signal propagates westwards during the evening hours (Fig. 2*F*), consistent with typical, long-lived MCS behavior. The limited westward propagation away from the Sierra Leone/Liberia border (Fig. 2*E*) is linked to the proximity of the coastline and its associated sea breeze, which is discussed later.

To provide a systematic analysis of deforestation impacts on convection, we analyzed core frequency trends in the vicinity of deforestation hotspots across the region. We used wavelet analysis to identify features with significant LST trends at different (East to West) length scales and composited core data on the center of each surface feature (see *Methods* and *SI Appendix*, Fig. 8). At all length scales sampled (16 to 194 km), we find systematically stronger trends in daily mean core frequency over and to the west of deforestation than to the east (Fig. 3). The daily East–West signal is dominated by trends during late afternoon and early evening. Local enhancements to the regionally positive core trend emerge clearly 1 h prior to the diurnal peak. This statistically significant signal propagates westward over the subsequent 2 h.

This analysis demonstrates that areas which have experienced strong deforestation since the 1990s have stronger convective trends during late afternoon than the surrounding regions. The location of enhanced afternoon storm initiation is consistent with mesoscale circulations driven by increased postdeforestation sensible heat flux (11). At larger deforestation length scales, the impact of reduced surface roughness in deforested areas may also enhance convergence and storm triggering (19). Interestingly, we find no evidence for a suppression effect over more extensive deforestation, in contrast to Amazonian studies (14, 19). In fact, in SWA, convective enhancement is stronger at larger length scales (84 and 194 km), suggesting that extensive deforestation is more effective at enhancing convective storm frequency. The nature and diurnal cycle of convection in SWA may play a key role in this length-scale behavior, as many late afternoon storms transform into longer-lived, westward-propagating MCSs. Indeed, a recent study (33) showed that anomalous surface warming on length scales ∼200 km is particularly effective at intensifying West African MCSs. In that analysis, the effect of reduced evaporation on low-level humidity was more than offset by enhanced moisture convergence, driven by surface heating. Considering the current study, idealized numerical modeling of MCSs is needed to quantify the relative importance of reduced surface evaporation versus enhanced moisture convergence and their sensitivities to the spatial scale of deforestation.

Finally, we consider the specific impact of deforestation near the coast and its potential to modify sea breeze convection. In Fig. 4, datasets have been composited as a function of perpendicular distance to the coastline, sampling the entire region from 15° W to 10° E. Climatologically, the sea breeze drives a maximum in convective core frequency parallel to the coast, which propagates inland during the afternoon (Fig. 4*A*). The seasonality of this convective signal corresponds closely to that of sea breeze observations along the Guinea Coast (35), with a clear minimum in both sea breeze days and core frequency during July and August (*SI Appendix*, Fig. 4*E*). Averaged along the entire coastline, the afternoon core frequency trend is positive (*P* < 0.05 for all inland distances from 1400 LT), reaching a maximum of 60% (relative to the 1982 to 1990 mean) some 20 to 40 km inland at 1530 LT. However, when only sampling longitudes with strong deforestation-driven LST trends within the 50-km coastal strip (see *Methods* and *SI Appendix*, Fig. 8*F*), the peak enhancement exceeds 100% (Fig. 4*B*) around 35 to 60 km inland, where tree cover loss is also maximized (Fig. 4*H*). By contrast, considering locations with weak LST trends, the core frequency trend is muted (Fig. 4*C*). This implies that deforestation in the coastal zone has a particularly strong impact on the frequency of afternoon convective storms via sea breeze processes. Warmer postdeforestation daytime temperatures enhance land–sea heating contrasts and thereby strengthen sea breezes. Reduced land surface roughness may also aid inland penetration of the breeze. In the presence of humid oceanic air, coastal deforestation is therefore more likely to trigger deep convection.

It is worth noting that within the coastal strip, the overall contribution of deep convective cores to intense rain rates decreases somewhat (*SI Appendix*, Fig. 5), consistent with other studies (26, 36). However, the deforestation–sea breeze effects we infer from Fig. 4 will also increase the frequency of shallower convective storms (warmer than −40 °C), which are not captured by our methodology. Moreover, the frequency of intense rainfall within the coastal belt measured by spaceborne radar follows the climatological diurnal cycle depicted in Fig. 4, with 30% of all intense rainfall occurring within the afternoon period (1400 to 1930 UTC).

Our sample of locations with coastal deforestation includes the capital cities of Conakry, Freetown, and Monrovia (*SI Appendix*, Fig. 8*F*), although urban pixels were removed before computing LST trends. The presence of these cities increases deforestation pressure nearby. In cities across SWA, adequate drainage networks are often lacking, particularly in their informal settlements. For urban populations already exposed to flash flooding, these risks are increased when accompanied by deforestation in the catchment. Removal of woody vegetation increases the runoff response to intense rainfall and can result in mud slides, as in the tragic event in Freetown in August 2017, where an estimated 1,100 people died (37). Our analysis shows that the deforestation-driven, local increase in convective storms is particularly marked precisely where exposure to flood risk is highest (Fig. 4 *B* and *K*). This illustrates a second, and previously unrecognized, impact of deforestation on flooding, as experienced in some of the least resilient cities on Earth. Planners should therefore consider the hydrometeorological impacts of continued deforestation in the regions surrounding major population centers.

## Discussion

Analysis of trends in convective storms across SWA has revealed a clear signature of deforestation at the mesoscale—patchy deforestation enhances the initiation of convection. The observations are consistent with forcing by thermally driven mesoscale circulations, as shown in previous modeling studies (11, 13) and observations from Amazonia (14, 15, 17). The localized nature and characteristic late afternoon timing of the signal provides a means to isolate the impact of deforestation from larger-scale storm trends. While in Amazonia rainfall is suppressed over larger deforested areas, we find the greatest increases in convective activity over more extensive regions of forest loss. This likely reflects a stronger contribution of long-lived MCSs to rainfall totals in SWA (38). Storm initiation is favored on the downwind edges of deforested patches by both thermal (18) and mechanical (19) mechanisms, which in Fig. 3 corresponds to the eastern side. Some of these storms will organize into westward-propagating MCSs (steered by the midlevel easterlies rather than low-level south westerlies) and bring enhanced convective activity right across the deforested zone and potentially hundreds of kilometers further west. Fig. 2*F* provides a specific example of this westward propagation, while previous West African analyses have illustrated the strong, positive effect on MCS rainfall of structures with enhanced, sensible heat flux (33, 39).

Like the majority of tropical deforestation hotspots, our study zone lies within a day’s advection time from the ocean (432 km assuming an inland wind speed of 5 ms^−1^). In this situation, plentiful atmospheric moisture supplied by the nearby ocean may suppress evaporation-driven responses of rainfall to deforestation and thereby enhance the relative role of dynamic mechanisms. One key dynamical process is the sea breeze, which is the dominant driver of convection over a number of deforested zones in SWA. Warmer postdeforestation temperatures enhance land–sea thermal contrasts and will produce more frequent convection embedded within the sea breeze. Increased storm frequency is particularly strong in the densely populated coastal strip of SWA, where vulnerability to climate-related flood hazards is already high. A similar combination of coastal deforestation and rapidly growing urban areas may also be changing exposure to flood risk in Southeast Asia, where more than a third of deforestation is occurring within 50 km of the coastline (*SI Appendix*, Fig. 1).

Deforestation throughout the 20th century means that the fraction of intact forest in SWA is far less than in Amazonia. Interestingly, the weak, coastal deforestation composite in Fig. 4 is made up predominantly of locations which were likely forest covered at the beginning of the 20th century (*SI Appendix*, Fig. 8*F*; ref. 20). By the end of that century, tree cover there was lower and population density higher than the SWA average (Fig. 4 *I* and *L*). Considering the 1982 to 1990 climatology, a location within the coastal belt included in the weak deforestation composite would experience afternoon (1400 to 1930 UTC) convective cores on 6.5 d/y. That compares with only 4.1 d/y for the deforestation sample, which, near the coast, was still relatively well-forested in 2000 (Fig. 4*H*). Over the last 30 y, the contrast in afternoon core frequency between the weak and strong deforestation composites has narrowed from 2.4 to 1.1 d/y. This suggests that the trends we have observed over the last 30 y are a manifestation of deforestation–sea breeze interactions that have been ongoing throughout the 20th century along the coastline of SWA. We are thus witnessing the impact on heavy rainfall of the late stages of deforestation in West Africa, quite unlike the current situation in Amazonia.

## Materials and Methods

This study uses a variety of datasets derived from satellite observations (Table 1). The primary data used in this study are cloud-top temperature images provided by the geostationary Meteosat series of satellites from the Eumetsat archive (https://www.eumetsat.int/). In 2004, the Meteosat First Generation (MFG) (40) of satellites was superseded by Meteosat Second Generation (MSG) (41). The Meteosat Visible and Infrared Imager onboard MFG satellites provided images in the thermal infrared (10.5 to 12.5 μm) every 30 min at a resolution of ∼4.5 km at the equator. We convert the data from counts to brightness temperature using coefficients provided by Eumetsat (https://www.eumetsat.int/mfg-calibration). For MSG, the Spinning Enhanced Visible and Infrared Imager provides higher spatial and temporal resolution (about 3 km and every 15 min) from 2004 onwards. We use MSG channel 9 data, centered at 10.8 μm.

**Table 1.** Satellite datasets used

Variable	Satellite(s)	Years available	Months used
Convective cores	MFG/MSG	1982–2020	March–November
Radar rainfall rate	TRMM	2004–2013	March–November
Land surface temperature	MODIS Terra	2001–2021	January–February
Land surface temperature	MODIS Aqua	2003–2021	January–February
Land surface temperature	MFG/MSG	1991–2015	January–February
Vegetation optical depth	Multiple	1991–2015	January–February
Forest cover change	Landsat	2000–2019	Annual
Land use land cover	Landsat	1975, 2000, 2013	Annual

Our analysis is based on the observed frequency of convective cores, which are identified from a two-dimensional wavelet scale decomposition of cloud-top temperature images (32). Each image is first regridded to a common spatial resolution of ∼5 km (0.045° regular grid), from which areas of contiguous cold (−40 °C or less) cloud larger than 700 km^2^ are extracted. We then filter the image for cloud features of length scales between 25 to 50 km in physical space using a Marr wavelet (42). We identify subcloud features within the 25 to 50 km scale range as contiguous areas of high power. Only nonzero power values exceeding the 25th centile threshold are defined as convective core areas. The frequency of convective cores is recorded at each pixel on the common grid for every 30-min slot in the day in each month, and annual data are based on the average of months March through November. Linear regressions and trends are computed from this annual dataset. The time of peak activity during the afternoon/evening period (1100 to 2000 LT; *SI Appendix*, Fig. 4) for a given pixel is determined from the full dataset (1982 to 2020) for the March to November period.

We use Level 2 data from the Tropical Rainfall Measurement Mission (TRMM) precipitation radar (43) to evaluate the statistical relationships between convective cores and rainfall. These are based on matching up instantaneous rainfall rates from TRMM overpasses with convective cores observed by Meteosat (as in ref. 32). Over SWA, we find that 80% of cores contain TRMM pixels flagged as convective rainfall. *SI Appendix*, Fig. 5*A* illustrates the frequency distribution of TRMM radar rain rates and the frequency with which that rate is contained within a core. The likelihood of light rain (<5 mm ⋅ h^−1^) occurring within a core is only 28.1%, but this number rises to 64.2% for intense rain (>20 mm ⋅ h^−1^). This association of cores with intense rain falls slightly near the coast (*SI Appendix*, Fig. 5*B*), consistent with an increasing contribution of warm rain there (26).

Previous studies have identified small biases and drifts associated with individual sensors in the Meteosat series but no systematic, long-term trend (44). Compared to a temperature threshold method, our wavelet approach minimizes sensitivity to instrumental biases (typically ∼1 K), as it identifies spatial variations in cloud-top temperature ∼4 to 12 K. Interannual variability in core frequency dominates over any instrumental artifacts (*SI Appendix*, Fig. 4*D*), and for the transition from MFG to MSG, we found no systematic changes in core frequency during the overlap period of 2004 to 2005.

The lack of stable, long-term precipitation datasets over SWA prevents us from accurately quantifying how trends in rainfall are influenced by deforestation. One important reason for this is the sparsity (31) and rapid degradation (45) of the rain gauge network in SWA. Considering annual mean rainfall, this contributes to the lack of agreement even on the sign of the long-term trend in different parts of the region (46). Estimating precipitation at higher space and time resolution, as required here, is considerably more challenging, and datasets with sufficient spatial and temporal resolution are typically only available from the beginning of the 21st century. Perhaps the most widely used of these is the Integrated MultisatellitE Retrievals for Global Precipitation Measurement (IMERG; refs. 47 and 48)]. The IMERG algorithm combines precipitation estimates from passive microwave imagery with subhourly infrared data from geostationary satellites such as Meteosat to fill in the gaps between microwave overpasses.

The major problem we found when generating subdiurnal trends in IMERG rainfall over its 20-y record is illustrated in *SI Appendix*, Fig. 6. For any given time of day, the availability of HQ precipitation estimates from passive microwave can change markedly from year to year, as new satellites are launched, and the local overpass time of satellites may drift substantially. This is reflected in changes in the mean annual weight used for merging infrared (IR) and microwave data. At 1630 LT (*SI Appendix*, Fig. 6*A*), on average only 2.4% of days have HQ data from 2001 to 2005, and the IR weighting is consequently large (∼0.6). Later in the dataset, there is increased availability of HQ data. For example, in 2020, on average, there is a suitable microwave overpass 35% of the time, and the annual IR weighting drops to ∼0.1. As a result, the combined precipitation estimate closely follows the rate based on IR imagery in the early years and follows the HQ data when the weighting is much lower. This is problematic in terms of computing trends, as the IR estimate at this time of day tends to be larger than the HQ value. The decreasing IR weighting over the 20 y contributes substantially to the negative trend in IMERG precipitation. We have quantified this effect of changes in availability of HQ data on IMERG trends for all times of day by multiplying the difference in long-term mean rainfall rates between IR and HQ with the annual IR weighting (*SI Appendix*, Fig. 6*B*). About 50% of the diurnal variation in IMERG rainfall trends can be explained by this artifact introduced by changes in the satellite constellation (*SI Appendix*, Fig. 6*C*). The effect is particularly important during the afternoon, corresponding to the time when deforestation has the strongest impact on convection.

Though the above artifact rules out combining IMERG trends from different times of day, in *SI Appendix*, Fig. 7 we compare trends in core frequency and IMERG rainfall estimates at 1830 LT for the period 2001 to 2020. This time is chosen specifically as it combines a high number of microwave images (average 30%) with a negligible trend in that number over the 20-y period. Despite the fundamental difference between the two measures (core trends provide a measure of changing storm frequency rather than rainfall rate), at this time of day, there is a clear, positive correlation between the two measures. Note that the correlation with the trend in the calibrated product is slightly weaker (*r* = 0.32, *P* < 0.001) than the uncalibrated rain rate, because of substantial differences over Ghana and southern Nigeria. We assume that this is due to changes there in the rain gauge network over time, which will introduce further local, artificial trends.

Trends in LST provide a direct measure of changes in the land surface energy budget. Large-scale, long-term LST trends are dominated by global warming. However, we focus on strong mesoscale variations, which highlight changes in land surface properties (notably the ability of the surface to evaporate and its aerodynamic roughness). We used three independent dry season (January/February) LST datasets. The Moderate Resolution Imaging Spectrometer on board the Terra and Aqua satellites provides two of these time series. We used the monthly 0.05° resolution LST products (MOD11C3 and MYD11C3 version 6; ref. 49) from 2001 and 2003, with equatorial overpass times of 1030 (Terra) and 1330 (Aqua) Local Time. A Climate Data Record derived from Meteosat imagery extends back to 1991 at 0.05° resolution (34, 50). We averaged hourly Meteosat LST data between 1200 and 1600 UTC to create monthly means. We found highly consistent mesoscale warming patterns from the three datasets during the overlap period. We created a single, annual LST time series from 1991 to 2021 at 0.05° resolution, based on averaging January and February data. Before averaging LST across datasets, we removed the mean LST from each satellite to minimize effects due to different sampling time, resolution, and sensor characteristics.

To justify our use of strong LST trends as a proxy for deforestation, we compared the data with three independent datasets, available over different time periods and/or spatial resolution. First, we used globally derived maps of forest cover change (2, 51) available from 2000 onward. We aggregated the 30-m pixels to create a forest loss (percent) dataset at 0.05°. Second, we compared dry season trends in LST and VOD at 0.25°. This microwave-based observation is linearly related with woody cover when the soil is dry (24), and we used Ku band data from 1991 to 2015 (52). Direct comparison of LST trends across the region produce negative correlations with VOD and tree cover trends (*P* < 0.00001).

Finally, we compared LST trends with changes identified from a series of West Africa–specific LULC maps (21, 53). Using Landsat imagery, the maps classify the region into 30 classes (including “Forest,” “Degraded Forest,” “Woodland,” “Savanna,” “Agriculture,” and “Settlements”) at 2-km resolution for the years 1975, 2000, and 2013. Net changes in the four wooded classes for the period 1975 to 2013 are shown in *SI Appendix*, Fig. 2 at 0.05° resolution. Their losses contributed primarily to agricultural extensification, which, over SWA, increased by a factor of 2.3 to 21.1% of land area.

Trends in these datasets are shown in *SI Appendix*, Fig. 3 as a function of LST trend over different periods, based on data availability. There is a strong sensitivity of LST trends to forest loss according to both the US Geological Survey (USGS) classification and the global dataset. Pixels losing the USGS-degraded forest class also contribute to intermediate (0.8 to 1.6 K ⋅ decade^−1^) warming, while trends in VOD and LST are well-correlated across the full range of observations. We conclude that once expanding urban settlements are removed, pixels with strong LST trends provide a good proxy for deforestation.

To illustrate the impact of deforestation on core frequency in specific hotspot areas (Fig. 2), we constructed annual, LST-based indices. From visual inspection of combined LST trends in each area, a threshold value was chosen to split pixels into either “deforested” or “nondeforested” samples. The index time series was then computed from the difference between the means of the two samples for each year and smoothed using a 5-y running mean. This approach was taken to reduce the impact of larger-scale fluctuations, trends, and satellite artifacts in dry season LST. In Fig. 2 *E* and *F*, convective core data have been regressed against the time series of LST indices (Fig. 2 *E* and *F*, *Insets*) for each pixel and time of day and are presented as the change in core frequency between the minimum and maximum of the LST indices.

The sensitivity of convective core frequency to deforestation is generalized to all locations (Fig. 3) by compositing cloud data on East–West transects across significant features in the LST trend field. These features are identified with a 1D Marr-wavelet scale decomposition in the zonal direction applied to the LST trend map, yielding centered local power maxima (LPM) for warm patches associated with physical length scales from 16 to 194 km (*SI Appendix*, Fig. 8 *A*–*D*). In Fig. 3, means and trends in core frequency are sampled as a function of East–West distance from each LPM, with the number of LPM ranging from 317 (at 16 km) to 81 (at 194 km). To provide an assessment of statistical significance, we compared deviations from the zonal mean trend (relative to climatology) against a second (“control”) set of core statistics sampled from across the region. We sampled the control statistics every 2° longitude by 1° latitude (*SI Appendix*, Fig. 8*E*) so that each sample pixel (dark blue) is spatially independent from its related LPM (pale blue), and the spatial correlation effect from compositing LPMs at adjacent latitudes is implicitly accounted for in the control sample. We calculated the 90% significance level from the 90th centile of the control set of statistics.

The analysis of convective cores affected by sea breezes (Fig. 4) is achieved by compositing fields perpendicular to the coastline at a resolution of 0.05°. Two subset composites are created, which represent strong and weak deforestation (locations shown in *SI Appendix*, Fig. 8*F*). The “coastal deforestation” composite (Fig. 4*B*) includes only coastal pixels in which LST trends exceed a threshold when averaged over the first 50 km. Coastal pixels are included in the “weak deforestation” sample (Fig. 4*C*) only when there are no 50-km inland sections in which LST trends exceed the threshold within at least 200 km. A value of 0.9 K ⋅ decade^−1^ was chosen as it provides a sufficiently large sample for robust averaging. Additional variables included in this analysis are population density estimates for 2000 and 2020 [ref. 54; available at 2.5 arc-minutes resolution (55)] and trends in March to November mean SST from ERA-5 reanalysis (56).

## Supplementary Material

Supplementary File

## Data Availability

All study data are included in the article and/or *SI Appendix*. All datasets used in this study are publicly available from the following sources: http://www.eumetsat.int (Meteosat), https://disc.gsfc.nasa.gov/datasets/TRMM_2A25_7/summary (precipitation radar), https://gpm.nasa.gov/data/imerg (IMERG), https://lpdaac.usgs.gov/products/mod11c3v006 and https://www.cmsaf.eu/EN/Home/home_node.html (LST), https://earthenginepartners.appspot.com/science-2013-global-forest (forest cover change), https://zenodo.org/record/2575599#.XhyeLSPLc-W (VOD), https://eros.usgs.gov/westafrica (Land Use/and Cover), https://sedac.ciesin.columbia.edu/data/collection/gpw-v4 (population), and https://climate.copernicus.eu/climate-reanalysis (SST).
