# Supplementary material for: “Late-stage” deforestation enhances storm trends in coastal West Africa
Source: Proc Natl Acad Sci U S A. 2022 Jan 4;119(2):e2109285119. doi: 10.1073/pnas.2109285119 (PMC8764663; doi:10.1073/pnas.2109285119)
Supplement: Supplementary File [file pnas.2109285119.sapp.pdf]

PNAS
www.pnas.org

Supplementary Information for
"Late-stage" deforestation enhances storm trends in coastal West Africa

Christopher M. Taylor, Cornelia Klein, Douglas J. Parker, France Gerard, Valiyaveetil Shamsudheen Semeena, Emma J. Barton, Bethan L. Harris

Corresponding author Christopher Taylor
Email: cmt@ceh.ac.uk

This PDF file includes:

Figures S1 to S8
SI References

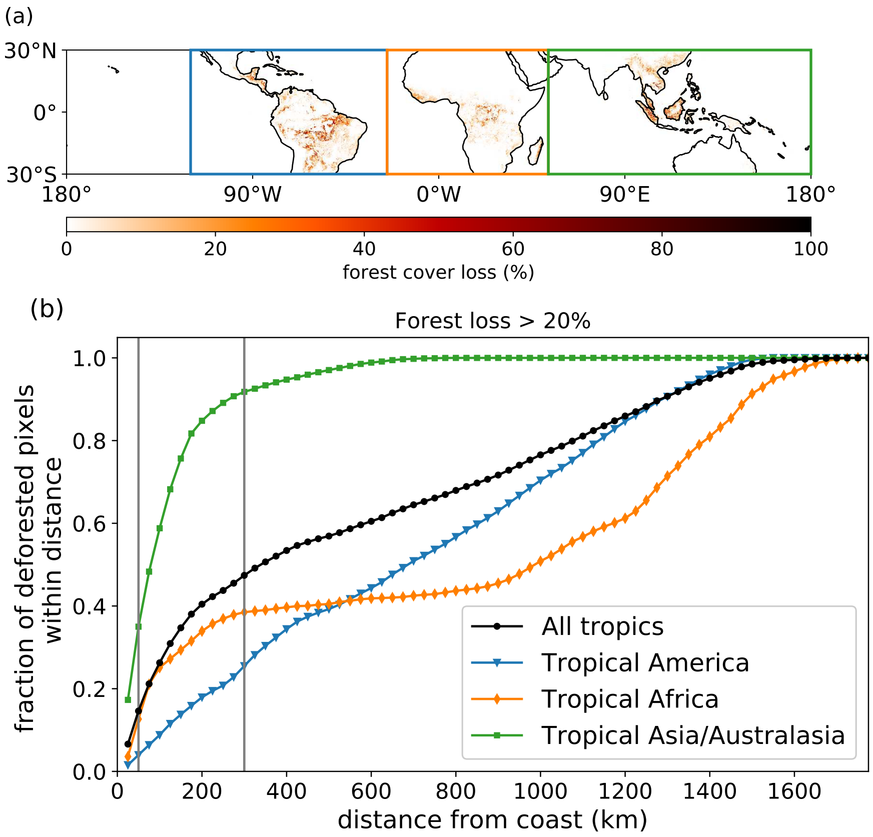

Fig. S1. Distribution of tropical deforestation and proximity to coastline. (a) Areas of deforestation and (b) cumulative distribution of deforestation as a function of distance from the coast. The coloured lines in (b) correspond to the areas shown by the coloured boxes in (a). Deforestation is based on forest loss between 2000 and 2019 (1) which exceeds 20% at the 0.05°scale. Vertical lines in (b) indicate 50 and 300 km from the coast.

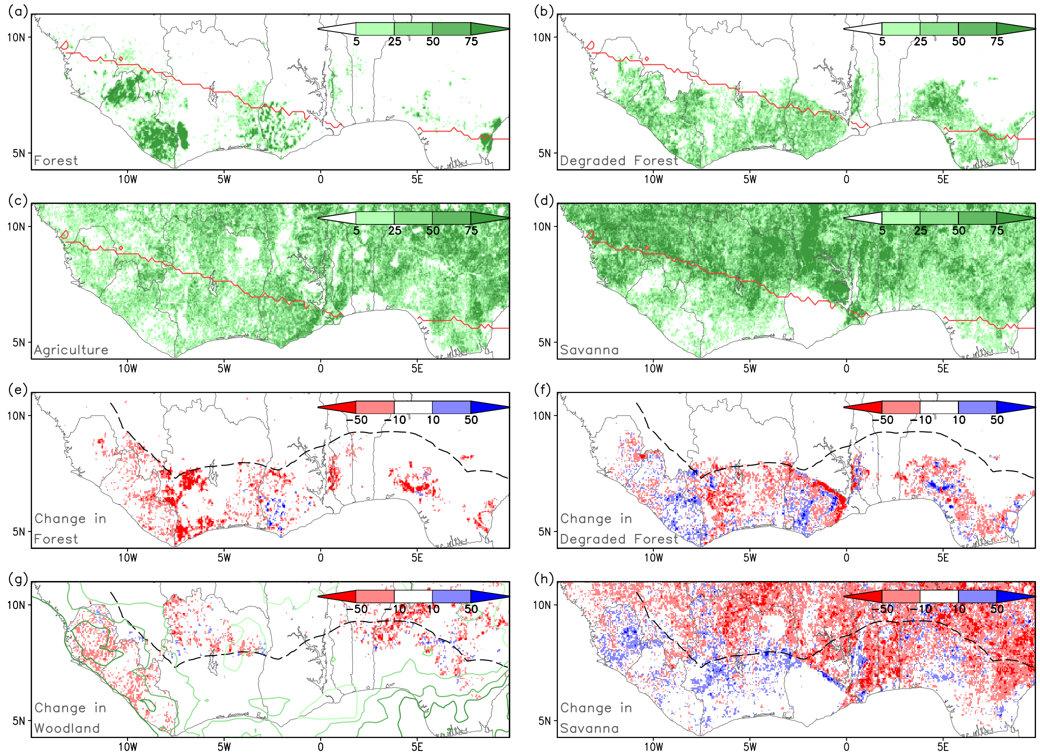

Fig. S2. Land use / land cover and its changes between 1975 and 2013 (2). Coverage [%] of the principal land types in 2013; (a) Forest, (b) Degraded Forest, (c) Agriculture, and (d) Savanna (including woody savanna). The red contour marks the estimated extent of forest cover in 1900 (3). The remaining figures show net changes in coverage of the 4 principal wooded classes over the 38 years to 2013; (e) Forest, (f) Degraded Forest, (g) Woodland, and (h) Savanna (including wooded savanna). The dashed black line denotes a distance of 300 km from the coast. All land cover data are plotted at a spatial resolution of 0.05°. In (g) the green contours show annual rainfall (1000, 1400, 1800 and 2200 mm, lighter to darker green) from the TRMM 3B43 dataset for the period 1998-2016. Note that the land use classification excludes Cameroon, in the southeast corner of the domain.

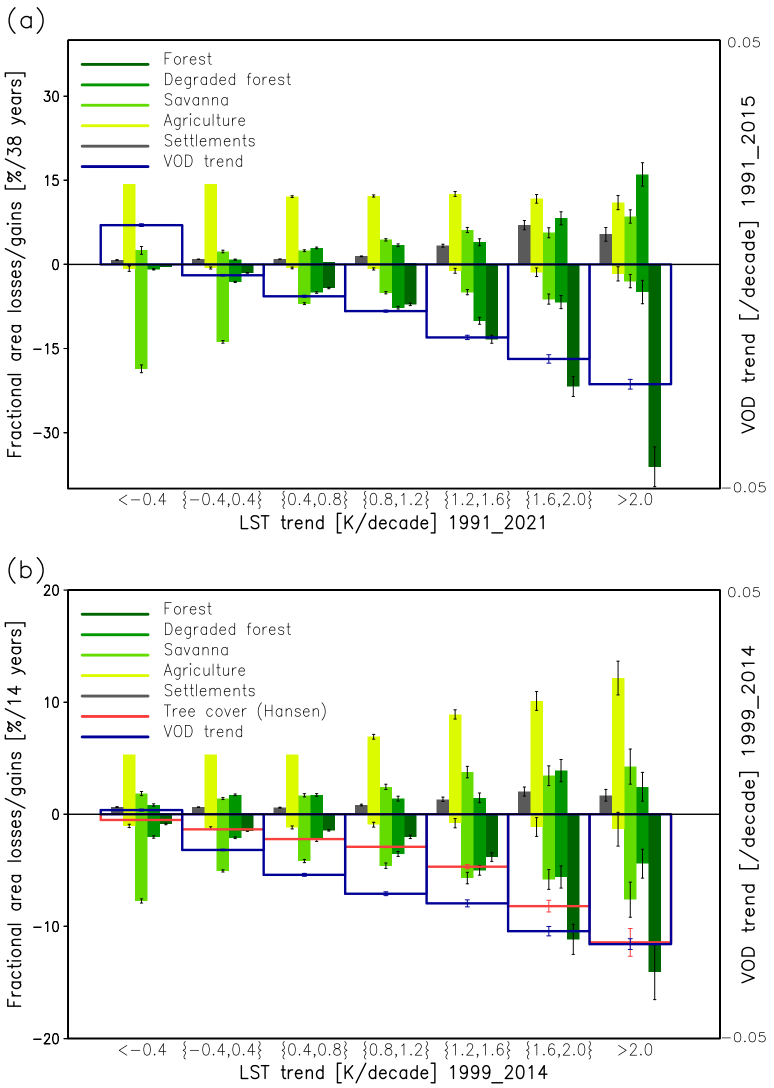

Fig. S3. Measures of deforestation as a function of LST trends. Changes in fractional area [%] covered by major LULC types (coloured bars) binned by LST trend [K decade^{-1}] for the periods (a) 1975-2013, and (b) 2000-2013. Gains and losses per 0.05°pixel are accounted for separately, and represented by bars above and below 0 respectively. Trends in vegetation optical depth VOD [decade^{-1}] for periods (a) 1991-2015 and (b) 1999-2014 are shown in blue, and in (b), tree cover trends for the period 2000-2013 shown in red. Standard errors on the mean changes/trends are depicted by error bars, and pixels further than 300km from the coast are excluded

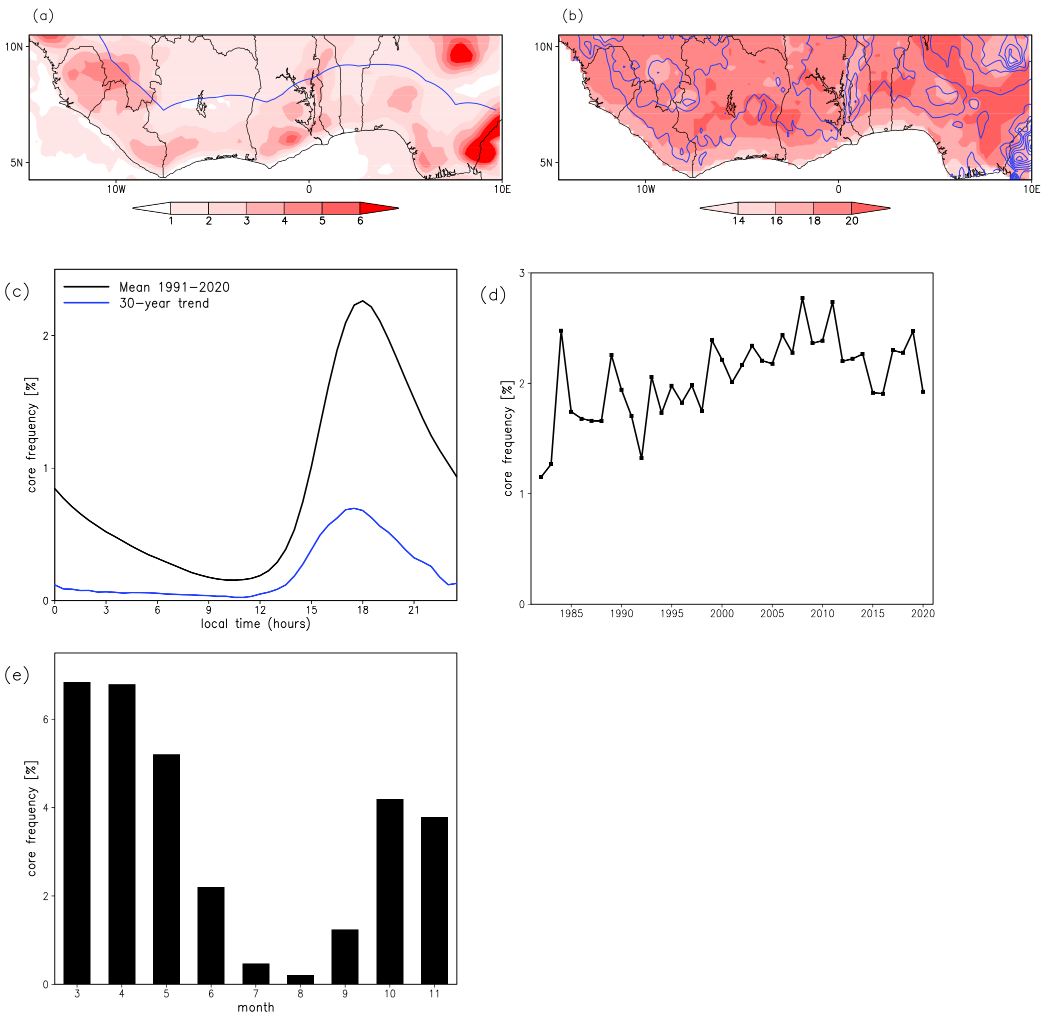

Fig. S4. Convective core characteristics. (a) Climatological mean core frequency at the afternoon maximum [%]. The blue contour denotes a distance of 300 km from the coastline. (b) Local time [hour] of afternoon maximum. Topographic height contours every 200 m are shown in blue. (c) Diurnal cycle of mean core frequency (black) and the change in frequency based on the 30-year trend (blue). (d) Annual evolution of core frequency sampled at 1800 LT. (e) Monthly mean frequency of convective cores 64 km inland from the Guinea Coast (4W-4E) at 1600 LT. In (c) and (d), data are averaged over the region within 300 km of the coastline.

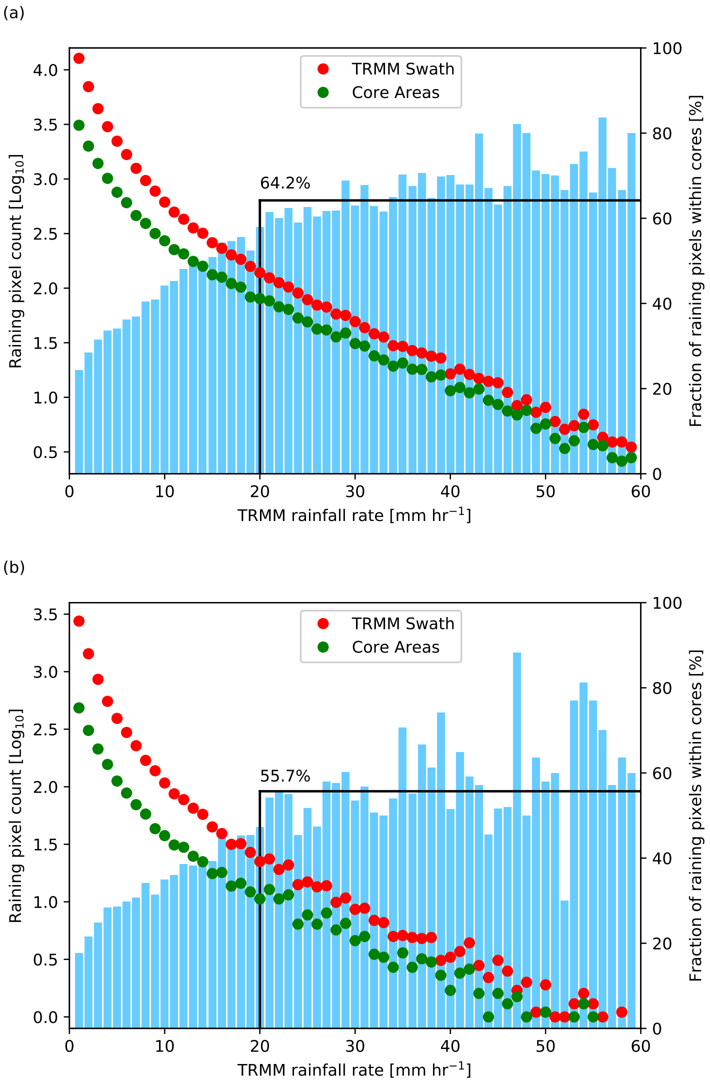

Fig. S5. Comparison of TRMM rainfall with convective cores. The frequency distribution of rainfall rates (binned every 1 mm hour^{-1}) from TRMM precipitation radar swaths over SWA (red circles). Green circles indicate the frequency of those raining pixels which occur within a core, also shown as a percentage of the TRMM count (blue bars). In (a), the data are taken from across SWA, and in (b), only data within 50 km of the coastline is used. On average, 64.2% (a) and 55.7% (b) of intense rainfall ($>$ 20 mm hour^{-1}) occurs within a convective core.

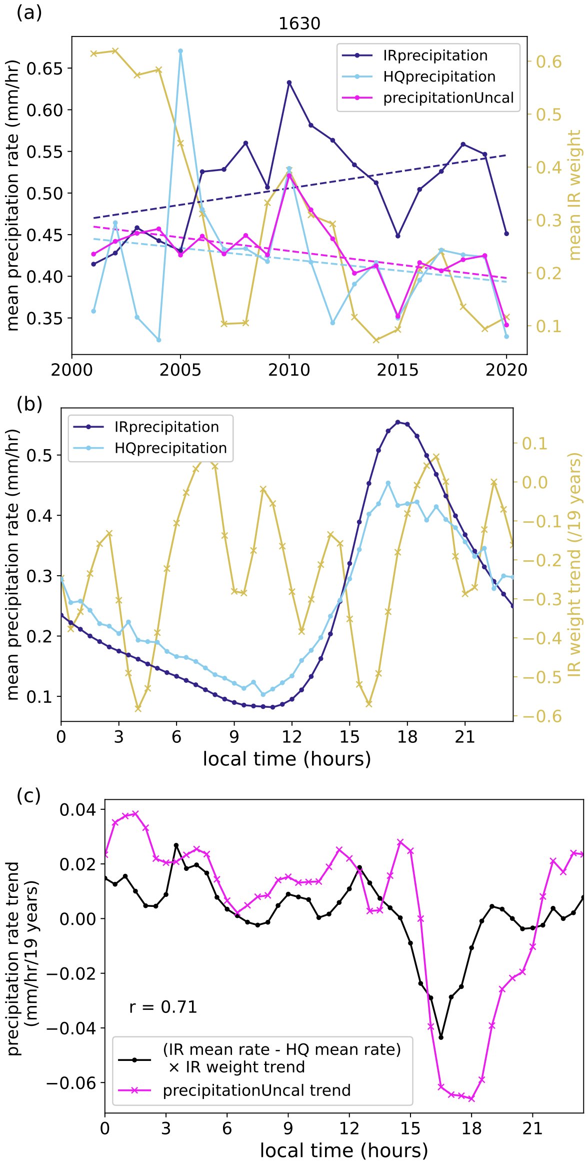

Fig. S6. Trends from IMERG. (a) Annual time series of IMERG precipitation rates (mm hour^{-1}) at 1630 LT estimated from infrared (IR; dark blue), microwave (HQ; pale blue) and the combined uncalibrated product (pink), with trends shown by dashed lines. The gold line depicts the annual mean weighting of the IR estimate. (b) Diurnal cycle of the long-term mean IR and HQ precipitation rates and the trend in IR weighting. (c) Diurnal cycle of the trend in the combined uncalibrated product (pink) and the artificial trend introduced by the trend in IR weighting (black). All calculations are averages over SWA within 300 km of the coastline, using data from March to November.

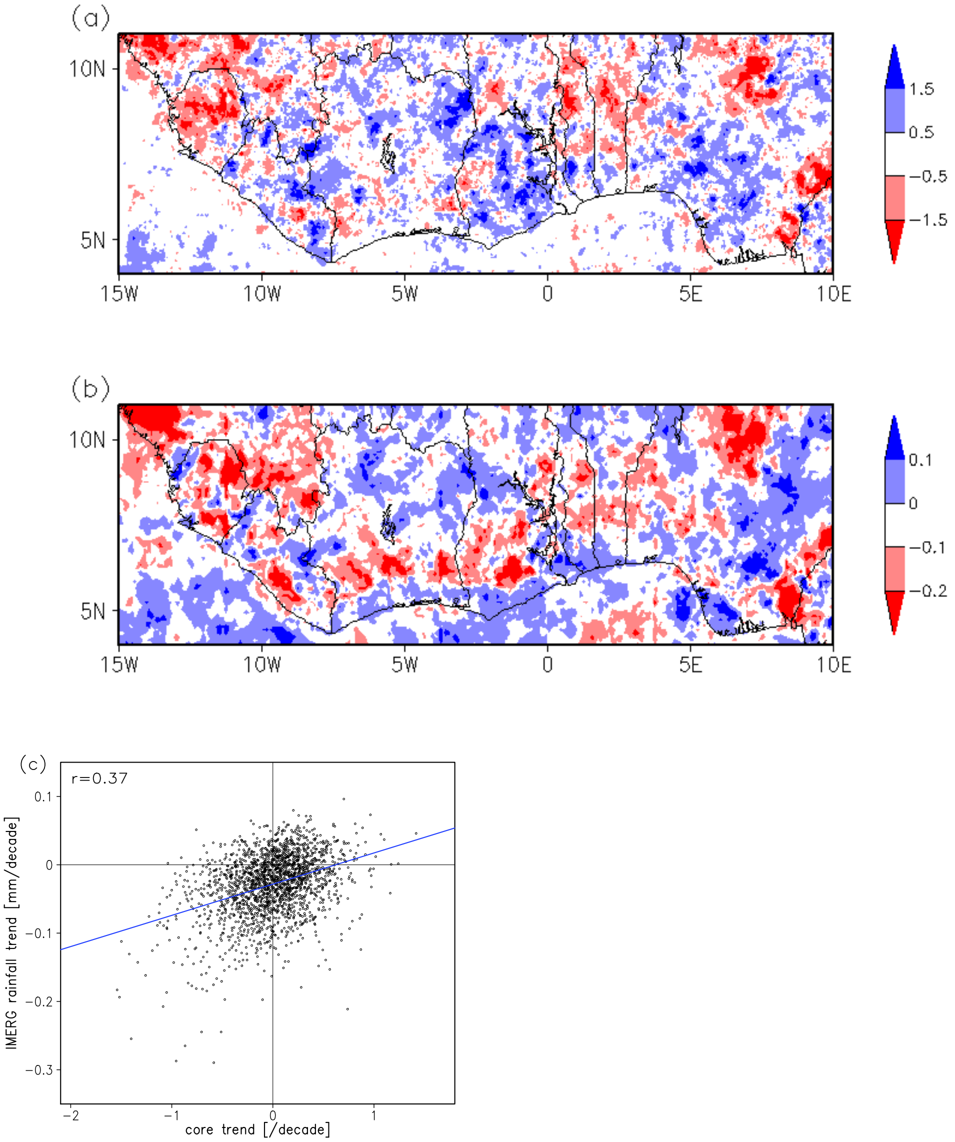

Fig. S7. Comparison of trends in core frequency and IMERG rainfall estimates. Trends in (a) core frequency (decade^{-1}) and (b) rainfall rate (mm hr^{-1} decade^{-1}) computed from data at 1830 LT for the months March-November for the period 2001-2020. In (c), trends in rainfall rate are plotted as a function of core frequency trends at a spatial resolution of 0.25 over land. The linear regression shown has a Pearson correlation coefficient of 0.37 (P<0.001).

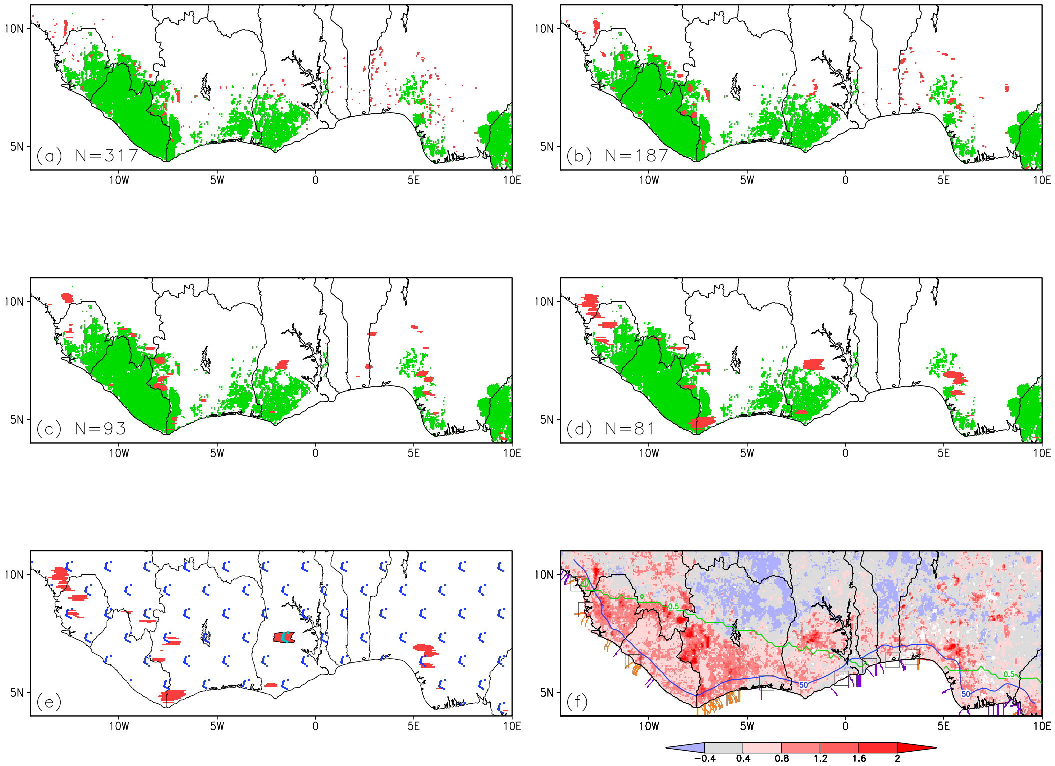

Fig. S8. Spatial sampling of composites. Locations of significant power in LST trend (red shading) used for compositing in Figure 3 at length scales of 16 (a), 37 (b), 84 (c) and 194 km (d and e). The number of patches sampled (N) is noted for each length scale. In (a)-(d), tree cover (1) exceeding 50% in 2000 is denoted by green shading. The sampling strategy for assessing statistical significance in Figure 3 is illustrated in (e) within an example deforestation region in central Ghana. Pale blue dots mark, for each latitude, the locations of Local Power Maxima used to centre core data, whilst dark blue dots illustrate locations used to build up a control sample on a regularly spaced grid. (f) Offshore locations used in the strong coastal warming (orange) and weak warming (purple) coastal composite in Figure 4. On the continent, shading depicts the LST trend (K decade^{-1})), and the green contour marks the extent of forest cover in 1900 (3). Principal cities in each country are shown by black squares.

SI References

1. Hansen MC*, et al.* (2013) High-Resolution Global Maps of 21st-Century Forest Cover Change. *Science* 342(6160):850-853.
2. CILSS (2016) *Landscapes of West Africa - A Window on a Changing World* (US Geological Survey, Ouagadougou, Burkina Faso) p 219.
3. Aleman JC, Jarzyna MA, & Staver AC (2018) Forest extent and deforestation in tropical Africa since 1900. *Nature Ecology & Evolution* 2(1):26-33.
